# Supplementary material for: Global earthworm distribution and activity windows based on soil hydromechanical constraints
Source: Commun Biol. 2021 May 21;4:612. doi: 10.1038/s42003-021-02139-5 (PMC8140072; doi:10.1038/s42003-021-02139-5)
Supplement: Supplementary file 2 — Supplemental Information [file 42003_2021_2139_MOESM2_ESM.docx]

Global earthworm distribution and activity windows based on soil hydromechanical constraints

Siul A. Ruiz^1,2*^, Samuel Bickel^1,3*^, and Dani Or ^1,4^

^1^Institute of Biogeochemistry and Pollutant Dynamics,

Soil and Terrestrial Environmental Physics, Swiss Federal Institute of Technology (ETH Zürich), Rämistrasse 101, 8092, Zürich, Switzerland

^2^Faculty of Engineering and Physical Sciences,

Bioengineering Group, University of Southampton, University Rd, Highfield, SO17 1BJ, Southampton, UK

^3^Institute of Terrestrial Ecosystems, Physics of Soils and Terrestrial Ecosystems, Swiss Federal Institute of Technology (ETH Zürich), Rämistrasse 101, 8092, Zürich, Switzerland

^4^Division of Hydrologic Sciences, Desert Research Institute, 2215 Raggio Pkwy, 89512, Reno, NV, USA

* These authors contributed equally to this work

**Name of corresponding author:** Siul Ruiz

**E-mail:** [s.a.ruiz@soton.ac.uk](mailto:siul.ruiz@usys.ethz.ch)

**Supplementary Information**

In this supplement, we provide additional information concerning the description of the biophysical, cavity-expansion model (SI.1), the functional dependency of hydromechanical properties with their parametrization (SI.2), the assessment of averaging methodology for summarizing earthworm limiting pressures (SI.3), evaluation of model predictions (SI.4), model sensitivity to changes in maximal earthworm pressure (SI.5), generalized analytic formulation for model sensitivity to perturbations in soil hydromechanical properties (SI.6), and a comparison between our mechanistic approach and two data-driven approaches for earthworm presence-only data (SI.7).

## **SI.1 – Cavity expansion mechanical model – an overview**

The mechanics of soil bioturbation by burrowing earthworms relies on their ability to deform the soil. The biophysical model used in this study considered soil penetration-cavity expansion sequences by the earthworm similar to cone penetration^11^. The model provided the minimal mechanical stress required to radially expand a cavity in an elasto-viscoplastic soil^11^. To quantify the magnitude of radial pressure required by an earthworm to expand in wet elasto-viscoplastic soils, we first considered the force balance at equilibrium:

|  | $\frac{\partial\sigma_{r}}{\partial r}+\frac{\sigma_{r}-\sigma_{\theta}}{r}=0$ | (S1) |
| --- | --- | --- |

where *r* [m] is the distance from the center of the cavity, $\sigma_{r}$ [Pa] is the radial stress and $\sigma_{\theta}$ [Pa] is the hoop (circumferential) stress. The deformation behavior is expressed by the Von-Mises criterion considering viscous deformation (i.e. Bingham model^24^), relating the difference between the radial and hoop stresses to the summation of the undrained soil strength and the viscoplastic strain rate:

|  | $\sigma_{r}-\sigma_{\theta}=2s_{u}+\frac{4}{3}\eta\dot{\epsilon}_{r}$ | (S2) |
| --- | --- | --- |

where $\eta$[Pa s] is the soil plastic viscosity, $s_{u}$ [Pa] is the undrained soil strength, and $\dot{\epsilon}_{r}$ [m m^-1^s^-1^] is the radial strain rate. Substitution of Eq. (S2) into (S1) yields the following expression:

|  | $\frac{\partial\sigma_{r}}{\partial r}=-\frac{2s_{u}}{r}-\frac{4}{3}\eta\frac{\dot{\epsilon}_{r}}{r}$ | (S3) |
| --- | --- | --- |

By integration, we determined the radial stresses as a function of the radius (and the strain rate):

|  | $\sigma_{r}\left( t,r \right)=P_{L}-2s_{u}ln\left( \frac{r}{r_{c}} \right)-\frac{4}{3}\eta\int\frac{\dot{\epsilon}_{r}}{r}dr$ | (S4) |
| --- | --- | --- |

where $r_{c}$ [m] is the minimum cavity size and $P_{L}$ [Pa] is the time-independent limit pressure to which the static cavity pressure converges. Under static conditions, the strain rate term in the integral vanishes. We solved for the limit pressure by equating the change in the cavity zone to the change in the plastic region local to the cavity:

|  | $\left( \frac{R_{p}}{r_{c}} \right)^{2}\to\frac{G}{s_{u}}$ | (S5) |
| --- | --- | --- |

where $G$ [Pa] is the soils shear modulus, and $R_{p}$ is the elasto-plastic interfacial radius. Under static conditions, the radial stress by the earthworm at the cavity wall is expressed as:

|  | $\sigma_{r}\left( R_{p} \right)=P_{L}-2s_{u}ln\left( \frac{R_{p}}{r_{c}} \right)=s_{u}$ | (S6) |
| --- | --- | --- |

leading to the minimum radial pressure required to expand a cavity in soil:

|  | $P_{L}=s_{u}\left( 1+2\ln\left( \frac{R_{p}}{r_{c}} \right) \right)= s_{u}\left( 1+\ln\left( \frac{G}{s_{u}} \right) \right)$ | (S7) |
| --- | --- | --- |

The resulting expression would be the minimum amount of pressure an earthworm would have to exert with its hydroskeleton to expand a cavity radially in soil. However, earthworms’ hydroskeleton is made up of soft flexible muscle fibers^27^ that are mechanically limited to a maximum pressure of $P_{w}$ = 200 kPa ^28,29^. Thus, earthworms are mechanically impeded by soil conditions when $P_{L}\geq P_{w}$. These constraining soil mechanical conditions are linked to soil’s hydration status and soil texture^45^.

## **SI.2 – Functional relationship between soil hydration status, textural class, and mechanical properties**

Soil mechanical properties are linked to multiscale physical phenomena, which are sensitive to soil textural class and soil moisture content. At the submicron scale, soil clay particles are tightly bound by electrical forces, and their ability to yield depends on their alignment, liquid lubrication, and platelet spacing^24^. The soil moisture plays a prominent role in binding together soil aggregates via capillarity under drier conditions^24, 25^ or by reducing soil friction under wetter conditions. Ultimately, these forces acting on different scales jointly increase soils shear strength and shear modulus of rigidity under drier conditions^11,26^. While these different processes warrant more rigorous analysis, these details extend beyond the scope of our current study.

Instead, we adopt simplified power-law relations for linking soil mechanical properties to soil texture and water content similar to the work of Gerard *et al.* (1965)^26^. We collected experimentally determined values for soil shear strength and shear modulus of rigidity and interpolated their behavior for a range of soil textures and soil water contents. Data were collected and consolidated from Gerard *et al.* (1965)^26^, Lu and Kaya (2013)^62^, Fan *et al.* (2017)^63^, Alramahi *et al.* (2010)^64^, Ruiz (2017)^11^, and Ghezzehei and Or (2001)^24^. Soil shear strength and shear modulus of rigidity is related to soil water contents via a power law ^26^:

|  | $s_{u}=a_{y}\left( f \right)\theta_{v}^{-b_{y}\left( f \right)}$ | (S8) |
| --- | --- | --- |
|  | $G=a_{G}\left( f \right)\theta_{v}^{-b_{G}\left( f \right)}$ | (S9) |

where $\theta_{v}$ [% m^3^ m^-3^] is the soil water content, the pre-factors $a_{y,G}(f)$ [Pa] and the exponent $b_{y,G}(f)$ [-] are functions of the soil fine fraction $f$ [% Silt+Clay]. Coefficients take the functional forms:

|  | $a_{y,G}\left( f \right)=\alpha_{y,G}exp(\beta_{y,G}f)$ | (S10) |
| --- | --- | --- |
|  | $b_{y,G}\left( f \right)=\zeta_{y,G}f+\xi_{y,G}$ | (S11) |

where $\alpha$, $\beta$, $\zeta$, and $\xi$ are fitting coefficients that relate the mechanical properties to the soil texture(i.e. the relationship with soil fine fraction). Lastly, the focus lies on the hydromechanical properties of relatively fine-textured soils, as coarse soils have been reported too abrasive and are often too dry for earthworm activity due to their frictional nature and low water retention properties respectively^45^.

## **Linking soil mechanical properties to soil texture and hydration state**

Coefficients and exponential pre-factors as related to soil fine texture content were plotted in Supplementary Fig. 1. A comprehensive span of soil shear strengths was taken from Gerard *et al.* (1965)^26^ and was parametrized using equations (S10) and (S11) (Supplementary Fig. 1 a and b). Equations (S10) and (S11) were also fitted to soil shear modulus data (Supplementary Fig. 1 c and d). These soil mechanical relations allowed us to determined minimal cavity expansion pressures via equation (S7) as a function of soil moisture contents and fine texture percentages (Supplementary Fig. 2). The red contour path in Supplementary Fig. 2 highlights soil conditions that inhibit earthworm mechanical activity. This functional relation was used to relate earthworm limiting pressures to soil moisture and soil texture.

## **SI.3 Assessing averaging methodologies for limiting pressures**

Soil moisture status is highly dynamic and limiting pressures respond non-linearly, thus it is not clear as to what averaging methods provide the most representative estimates for promoting potential earthworm habitats. We compared the model results considering arithmetic averaging, harmonic averaging, and median values of the global limit pressures that would support earthworm activity (Supplementary Fig. 4). To systematically compare the effect of the averaging method on predicted regions below the earthworm limiting pressure ($P_{w}$ = 200 kPa), we overlaid masks for each method (Supplementary Fig. 4 a-c) and counted the number of times predictions agree (Supplementary Fig. 4 d). Most regions are considered permissible to earthworm bioturbation by all three averaging methods. The arithmetic average resulted in more restricted regions, while the harmonic mean classified a larger proportion of the terrestrial surface as suitable habitats based on limit pressure. The difference between averaging methods was most pronounced in regions with larger monthly variability in soil moisture (e.g. Mediterranean, India, Sahel).

## **SI.4 Evaluating model predictions**

We evaluated our model predictions with four independent datasets. Global earthworm abundance data^20^ indicates only low abundance above the predicted pressure limit (Supplementary Fig. 5). Earthworm occurrence data^14^ was used to evaluate the number of true positive sites under resampling (Supplementary Fig. 7). The same data was used together with another global dataset^14^ to compare modeled thresholds to ranges of environmental conditions (Fig 2, Supplementary Fig. 6).

## **SI.5 Model prediction sensitivity to changes in maximal earthworm pressures**

Supplementary Fig. 3 shows that using a threshold of 200 kPa that was determined experimentally^12, 28, 29^ recovers almost 90% of global occurrences and indicates that the value represents an inclusive upper bound (covering the wide range of observed earthworm pressure limits). This is in agreement with earthworm abundance data from an independent study (Johnston et al, (2019)^20^; Supplementary Fig. 5) where around 90% of abundances occurred below 200 kPa. Given our observations on earthworm habitat distributions and abundance time series (Fig 4 c), a more conservative value of 100 kPa could be used operationally to delineate regions as earthworm habitats (and would correspond to around 75% of occurrences). Here we use the more inclusive, physically-based upper bound of 200 kPa.

## **SI.6 Formulation for model sensitivity to changes in hydromechanical properties**

As the limit pressure model is an analytic expression, we can analytically determine model sensitivity to input parameters. Using variational principles, we considered a perturbation to the limit pressure as follows:

|  | $\delta P_{L}=\left[ \begin{matrix} \frac{\partial P_{L}}{\partial s_{u}} & \frac{\partial P_{L}}{\partial G} \end{matrix} \right]\left[ \begin{aligned} \delta s_{u} \\ \delta G \end{aligned} \right]$ | (S12) |
| --- | --- | --- |

Where $\delta$ represents a small perturbation in a given parameter. Considering that the soil shear strength and the shear modulus are both functions of soil texture ($f$) and water content ($\theta_{v}$), eq (S12) expands to:

|  | $\delta P_{L}=\left[ \begin{matrix} \frac{\partial P_{L}}{\partial s_{u}} & \frac{\partial P_{L}}{\partial G} \end{matrix} \right]\left[ \begin{matrix} \frac{\partial s_{u}}{\partial\theta_{v}} & \frac{\partial s_{u}}{\partial f} \\ \frac{\partial G}{\partial\theta_{v}} & \frac{\partial G}{\partial f} \end{matrix} \right]\left[ \begin{aligned} \delta\theta_{v} \\ \delta f \end{aligned} \right]$ | (S13) |
| --- | --- | --- |

which simplifies to the following scalar expression:

|  | $\delta P_{L}=\left( \frac{\partial P_{L}}{\partial s_{u}}\frac{\partial s_{u}}{\partial\theta_{v}}+\frac{\partial P_{L}}{\partial G}\frac{\partial G}{\partial\theta_{v}} \right)\delta\theta_{v}+ \left( \frac{\partial P_{L}}{\partial s_{u}}\frac{\partial s_{u}}{\partial f}+\frac{\partial P_{L}}{\partial G}\frac{\partial G}{\partial f} \right)\delta f$ | (S14) |
| --- | --- | --- |

Substituting in the partial derivatives of eq. (7), we get:

|  | $\delta P_{L}=\left( \ln\left( \frac{G}{s_{u}} \right)\frac{\partial s_{u}}{\partial\theta_{v}}+\frac{s_{u}}{G}\frac{\partial G}{\partial\theta_{v}} \right)\delta\theta_{v}+ \left( \ln\left( \frac{G}{s_{u}} \right)\frac{\partial s_{u}}{\partial f}+\frac{s_{u}}{G}\frac{\partial G}{\partial f} \right)\delta f$ | (S15) |
| --- | --- | --- |

Substituting in equations (S8), (S9), (S10), and (S11), we get an expression without any partial derivatives:

|  | $\delta P_{L}=\left( \ln\left( \frac{G}{s_{u}} \right)\left( -\frac{a_{y}b_{y}}{\theta_{v}^{b_{y}+1}} \right)+\frac{s_{u}}{G}\left( -\frac{a_{G}b_{G}}{\theta_{v}^{b_{G}+1}} \right) \right)\delta\theta_{v}+ \left( \ln\left( \frac{G}{s_{u}} \right)\left( \frac{\left( \alpha_{y}e^{\beta_{y}f}\left( \beta_{y} - \zeta_{y}ln\left( \theta_{v} \right) \right) \right)}{\theta_{v}^{\xi_{y} + f\zeta_{y}}} \right)+\frac{s_{u}}{G}\left( \frac{\left( \alpha_{G}e^{\beta_{G}f}\left( \beta_{G} - \zeta_{G}ln\left( \theta_{v} \right) \right) \right)}{\theta_{v}^{\xi_{G} + f\zeta_{G}}} \right) \right)\delta f$ | (S16) |
| --- | --- | --- |

Completely expanded provides the final expression:

|  | $\delta P_{L}=\left( \ln\left( \frac{\alpha_{G}exp(\beta_{G}f)\theta_{v}^{-\left( \zeta_{G}f+\xi_{G} \right)}}{\alpha_{y}exp(\beta_{y}f)\theta_{v}^{-\left( \zeta_{y}f+\xi_{y} \right)}} \right)\left( -\frac{\alpha_{y}exp(\beta_{y}f)(\zeta_{y}f+\xi_{y})}{\theta_{v}^{(\zeta_{y}f+\xi_{y})+1}} \right)+\frac{\alpha_{y}exp(\beta_{y}f)\theta_{v}^{-\left( \zeta_{y}f+\xi_{y} \right)}}{\alpha_{G}exp(\beta_{G}f)\theta_{v}^{-\left( \zeta_{G}f+\xi_{G} \right)}}\left( -\frac{\alpha_{G}exp(\beta_{G}f)(\zeta_{G}f+\xi_{G})}{\theta_{v}^{(\zeta_{G}f+\xi_{G})+1}} \right) \right)\delta\theta_{v}+ \left( \ln\left( \frac{\alpha_{G}exp(\beta_{G}f)\theta_{v}^{-\left( \zeta_{G}f+\xi_{G} \right)}}{\alpha_{y}exp(\beta_{y}f)\theta_{v}^{-\left( \zeta_{y}f+\xi_{y} \right)}} \right)\left( \frac{\left( \alpha_{y}e^{\beta_{y}f}\left( \beta_{y} - \zeta_{y}ln\left( \theta_{v} \right) \right) \right)}{\theta_{v}^{\xi_{y} + f\zeta_{y}}} \right)+\frac{\alpha_{y}exp(\beta_{y}f)\theta_{v}^{-\left( \zeta_{y}f+\xi_{y} \right)}}{\alpha_{G}exp(\beta_{G}f)\theta_{v}^{-\left( \zeta_{G}f+\xi_{G} \right)}}\left( \frac{\left( \alpha_{G}e^{\beta_{G}f}\left( \beta_{G} - \zeta_{G}ln\left( \theta_{v} \right) \right) \right)}{\theta_{v}^{\xi_{G} + f\zeta_{G}}} \right) \right)\delta f$ | (S17) |
| --- | --- | --- |

In accordance with eq. (S16) or (S17), we consider standard deviations as system perturbations and examine the regions that are most volatile based on soil moisture status and texture.

## **SI.6 Comparison between mechanistic and data-driven approaches.**

Metrics that infer goodness of fit cannot be applied to our mechanistic framework, as our model is not fit to the earthworm occurrence data. In this section, we present a comparison between our mechanistic model and two data-driven approaches for earthworm presence-only data (gridded to 0.1° x 0.1°, n = 7156). The first method used is a one-class support vector machine (Drake *et al.*, 2006^65^) (oc-SVM, with parameters *η* = 0.1 and *γ* = 0.5), which is an unsupervised algorithm that outputs a decision function that indicates positive and negative classifications (i.e., occurrences). The second approach is based on the Mahalanobis distance (Etherington and Thomas, 2019^66^) (*D^2^*), which measures the distance of a point to the center of a multivariate normal distribution (a critical distance is found using chi-square statistic assuming a p-value of 0.01). Thus, occurrences are classified based on values that are inliers (or “within the niche space”). Both methods have been used in ecology as they closely relate to the concept of fundamental niches. To provide a non-mechanistic benchmark, we used the following covariates (values were scaled to their range for the oc-SVM): mean annual temperature, soil pH, sand content and mean annual precipitation. We then compared the resulting masks of habitat suitability (Supplementary Fig 8).

The masks obtained from the different methods are similar and differences in true positives are smaller than the proportion of occurrences at river corridors and in certain regions in the permafrost (~10%). However, we note that while a data-driven approach may provide similar results, our mechanistic model has the added benefit of explaining the physical principles underpinning the map. As such, data-driven approaches may lead to flawed or incomplete conclusions, for example, regarding the importance of soil properties and the estimation of tropical earthworm diversity and abundance (see Phillips *et al.* 2019^14^ with erratum and correspondences).

# **References**

1 Young, I. M. *et al.* The interaction of soil biota and soil structure under global change. *Global Change Biology* **4**, 703-712 (1998).

2 Lavelle, P. *et al.* Earthworms as key actors in self-organized soil systems. *Theoretical Ecology Series* **4**, 77-106 (2007).

3 Blakemore, R. & Hochkirch, A. Soil: Restore earthworms to rebuild topsoil. *Nature* **545**, 30-30 (2017).

4 Kuzyakov, Y. & Blagodatskaya, E. Microbial hotspots and hot moments in soil: Concept & review. *Soil Biology and Biochemistry* **83**, 184-199 (2015).

5 Brown, G. G., Barois, I. & Lavelle, P. Regulation of soil organic matter dynamics and microbial activityin the drilosphere and the role of interactionswith other edaphic functional domains. *European Journal of Soil Biology* **36**, 177-198 (2000).

6 Denef, K. *et al.* Influence of dry–wet cycles on the interrelationship between aggregate, particulate organic matter, and microbial community dynamics. *Soil Biology and Biochemistry* **33**, 1599-1611 (2001).

7 Van Groenigen, J. W. *et al.* Earthworms increase plant production: a meta-analysis. *Scientific reports* **4** (2014).

8 Blouin, M. *et al.* A review of earthworm impact on soil function and ecosystem services. *European Journal of Soil Science* **64**, 161-182 (2013).

9 Capowiez, Y. *et al.* Experimental evidence for the role of earthworms in compacted soil regeneration based on field observations and results from a semi-field experiment. *Soil Biology and Biochemistry* **41**, 711-717 (2009).

10 Wu, X. D., Guo, J. L., Han, M. & Chen, G. An overview of arable land use for the world economy: From source to sink via the global supply chain. *Land use policy* **76**, 201-214 (2018).

11 Ruiz, S., Schymanski, S. & Or, D. Mechanics and Energetics of Soil Penetration by Earthworms and Plant Roots - Higher Burrowing Rates Cost More. *Vadose Zone Journal* **10.2136/vzj2017.01.0021**, doi:10.2136/vzj2017.01.0021 (2017).

12 Quillin, K. J. Kinematic scaling of locomotion by hydrostatic animals: ontogeny of peristaltic crawling by the earthworm Lumbricus terrestris. *Journal of Experimental Biology* **202**, 661-674 (1999).

13 Ruiz, S., Or, D. & Schymanski, S. Soil Penetration by Earthworms and Plant Roots—Mechanical Energetics of Bioturbation of Compacted Soils. *PLOS ONE* **10.1371/journal.pone.0128914** (2015).

14 Phillips, H. R. *et al.* Global distribution of earthworm diversity. *Science* **366**, 480-485 (2019).

15 Abbott, I. Distribution of the native earthworm fauna of Australia-a continent-wide perspective. *Soil Research* **32**, 117-126 (1994).

16 Hendrix, P. F. & Bohlen, P. J. Exotic earthworm invasions in North America: ecological and policy implications. *Bioscience* **52**, 801-811 (2002).

17 Nakamura, Y. Studies on the Ecology of Terrestrial Oligochaeta: I. Sesonal Variation in the Population Density of Earthworms in Alluvial Soil Grassland in Sapporo, Hokkaido. *Applied Entomology and Zoology* **3**, 89-95 (1968).

18 Edwards, C. A. & Bohlen, P. J. *Biology and ecology of earthworms*. Vol. 3 (Springer Science & Business Media, 1996).

19 Kretzschmar, A. Burrowing ability of the earthworm Aporrectodea longa limited by soil compaction and water potential. *Biology and Fertility of Soils* **11**, 48-51 (1991).

20 Johnston, A. S. Land management modulates the environmental controls on global earthworm communities. *Global Ecology and Biogeography* **28**, 1787-1795 (2019).

21 Rao, K. P. Physiology of low temperature acclimation in tropical poikilotherms. I. Ionic changes in the blood of the freshwater mussel, Lamellidens marginalis, and the earthworm, Lampito mauritii. *Proceedings of the Indian Academy of Sciences-Section B* **57**, 290-295 (1963).

22 Baker, G. H. & Whitby, W. A. Soil pH preferences and the influences of soil type and temperature on the survival and growth of Aporrectodea longa (Lumbricidae): The 7th international symposium on earthworm ecology· Cardiff· Wales· 2002. *Pedobiologia* **47**, 745-753 (2003).

23 El-Duweini, A. K. & Ghabbour, S. I. Population density and biomass of earthworms in different types of Egyptian soils. *Journal of Applied Ecology*, 271-287 (1965).

24 Ghezzehei, T. A. & Or, D. Rheological properties of wet soils and clays under steady and oscillatory stresses. *Soil Science Society of America Journal* **65**, 624-637 (2001).

25 Ghezzehei, T. A. & Or, D. Dynamics of soil aggregate coalescence governed by capillary and rheological processes. *Water Resources Research* **36**, 367-379 (2000).

26 Gerard, C. The Influence of Soil Moisture, Soil Texture, Drying Conditions, and Exchangeable Cations on Soil Strength. *Soil Science Society of America Journal* **29**, 641-645 (1965).

27 Quillin, K. J. Ontogenetic scaling of burrowing forces in the earthworm Lumbricus terrestris. *Journal of Experimental Biology* **203**, 2757-2770 (2000).

28 Ruiz, S. A. & Or, D. Biomechanical limits to soil penetration by earthworms: direct measurements of hydroskeletal pressures and peristaltic motions. *Journal of The Royal Society Interface* **15**, 20180127 (2018).

29 McKenzie, B. M. & Dexter, A. R. Radial pressures generated by the earthworm Aporrectodea rosea. *Biology and Fertility of Soils* **5**, 328-332 (1988).

30 Hengl, T. *et al.* SoilGrids250m: Global gridded soil information based on machine learning. *PLoS one* **12**, e0169748 (2017).

31 Burges, A. *Soil biology*. (Elsevier, 2012).

32 Ruiz, S. A. Mechanics and Energetics of Soil Bioturbation by Earthworms and Growing Plant Roots. doi:10.3929/ethz-b-000280625 (2018).

33 Kretzschmar, A. & Bruchou, C. Weight response to the soil water potential of the earthworm Aporrectodea longa. *Biology and fertility of soils* **12**, 209-212 (1991).

34 Eggleton, P., Inward, K., Smith, J., Jones, D. T. & Sherlock, E. A six year study of earthworm (Lumbricidae) populations in pasture woodland in southern England shows their responses to soil temperature and soil moisture. *Soil Biology and Biochemistry* **41**, 1857-1865 (2009).

35 Beer, C., Reichstein, M., Ciais, P., Farquhar, G. & Papale, D. Mean annual GPP of Europe derived from its water balance. *Geophysical Research Letters* **34** (2007).

36 Keudel, M. & Schrader, S. Axial and radial pressure exerted by earthworms of different ecological groups. *Biology and Fertility of Soils* **29**, 262-269 (1999).

37 Heaney, L. R., Balete, D. S., Rickart, E. A. & Niedzielski, A. *The mammals of Luzon Island: biogeography and natural history of a Philippine fauna*. (Johns Hopkins University Press, 2016).

38 Keller, T. *et al.* Long-Term Soil Structure Observatory for Monitoring Post-Compaction Evolution of Soil Structure. *Vadose Zone Journal* **16** (2017).

39 Lacoste, M., Ruiz, S. & Or, D. Listening to earthworms burrowing and roots growing-acoustic signatures of soil biological activity. *Scientific reports* **8**, 10236 (2018).

40 Kearney, M. & Porter, W. Mechanistic niche modelling: combining physiological and spatial data to predict species’ ranges. *Ecology letters* **12**, 334-350 (2009).

41 IPCC. The Physical Science Basis. Contribution of Working Group I to the Fifth Assessment Report of the Intergovernmental Panel on Climate Change [Stocker, T.F., D. Qin, G.-K. Plattner, M. Tignor, S.K. Allen, J. Boschung, A. Nauels, Y. Xia, V. Bex and P.M. Midgley (eds.)]. 1535 (Cambridge University Press, Cambridge, United Kingdom and New York, NY, USA, 2013).

42 Van Den Hoogen, J. *et al.* Soil nematode abundance and functional group composition at a global scale. *Nature* **572**, 194-198 (2019).

43 Bengough, A. G. *et al.* Root responses to soil physical conditions; growth dynamics from field to cell. *Journal of Experimental Botany* **57**, 437-447 (2005).

44 Beer, C. *et al.* Terrestrial gross carbon dioxide uptake: global distribution and covariation with climate. *Science* **329**, 834-838 (2010).

45 Paoletti, M. G. The role of earthworms for assessment of sustainability and as bioindicators. *Agriculture, Ecosystems & Environment* **74**, 137-155 (1999).

46 Gruber, S. Derivation and analysis of a high-resolution estimate of global permafrost zonation. *The Cryosphere* **6**, 221 (2012).

47 Muñoz Sabater, J. (ed Copernicus Climate Change Service (C3S) Climate Data Store (CDS).) (2019).

48 Beck, H. E. *et al.* MSWEP V2 global 3-hourly 0.1° precipitation: methodology and quantitative assessment. *Bulletin of the American Meteorological Society* **100**, 473-500 (2019).

49 Chamberlain, E. J. & Butt, K. R. Distribution of earthworms and influence of soil properties across a successional sand dune ecosystem in NW England. *European Journal of Soil Biology* **44**, 554-558 (2008).

50 Booth, L. H., Heppelthwaite, V. & McGlinchy, A. The effect of environmental parameters on growth, cholinesterase activity and glutathione S-transferase activity in the earthworm (Apporectodea caliginosa). *Biomarkers* **5**, 46-55 (2000).

51 GBIF.org. GBIF Occurrence Download (Almidae). doi:<https://doi.org/10.15468/dl.xstqow> (2020).

52 GBIF.org. GBIF Occurrence Download (Eudrilidae). doi:<https://doi.org/10.15468/dl.wghggg> (2020).

53 GBIF.org. GBIF Occurrence Download (Glossoscolecidae). doi:<https://doi.org/10.15468/dl.3yj8pk> (2020).

54 GBIF.org. GBIF Occurrence Download (Hormogastridae). doi:<https://doi.org/10.15468/dl.lzuwlg> (2020).

55 GBIF.org. GBIF Occurrence Download (Lumbricidae). doi:<https://doi.org/10.15468/dl.vwqtsk> (2020).

56 GBIF.org. GBIF Occurrence Download (Microchaetidae). doi:<https://doi.org/10.15468/dl.brqmht> (2020).

57 GBIF.org. GBIF Occurrence Download (Moniligastridae). doi:<https://doi.org/10.15468/dl.ghccto> (2020).

58 GBIF.org. GBIF Occurrence Download (Ocnerodrilidae). doi:<https://doi.org/10.15468/dl.dk97gk> (2020).

59 GBIF.org. GBIF Occurrence Download (Octochaetidae). doi:<https://doi.org/10.15468/dl.xjw6kc> (2020).

60 GBIF.org. GBIF Occurrence Download (Sparganophilidae). doi:<https://doi.org/10.15468/dl.9a4ojx> (2020).

61 Ruiz, S. B., S; Or, D. Dataset for: GLOBAL EARTHWORM DISTRIBUTION AND ACTIVITY WINDOWS BASED ON SOIL HYDROMECHANICAL CONSTRAINTS. doi:<https://doi.org/10.3929/ethz-b-000476615> (2021).

62 Lu, N. & Kaya, M. Power law for elastic moduli of unsaturated soil. *Journal of Geotechnical and Geoenvironmental Engineering* **140**, 46-56 (2014).

63 Fan, L., Lehmann, P. & Or, D. Load redistribution rules for progressive failure in shallow landslides: Threshold mechanical models. *Geophysical Research Letters* **44**, 228-235 (2017).

64 Alramahi, B., Alshibli, K. A. & Fratta, D. Effect of fine particle migration on the small-strain stiffness of unsaturated soils. *Journal of geotechnical and geoenvironmental engineering* **136**, 620-628 (2010).

65 Drake, J. M., Randin, C. & Guisan, A. Modelling ecological niches with support vector machines. *Journal of applied ecology* **43**, 424-432 (2006).

66 Etherington, T. R. Mahalanobis distances and ecological niche modelling: correcting a chi-squared probability error. *PeerJ* **7**, e6678 (2019).

**Supplementary Figures**


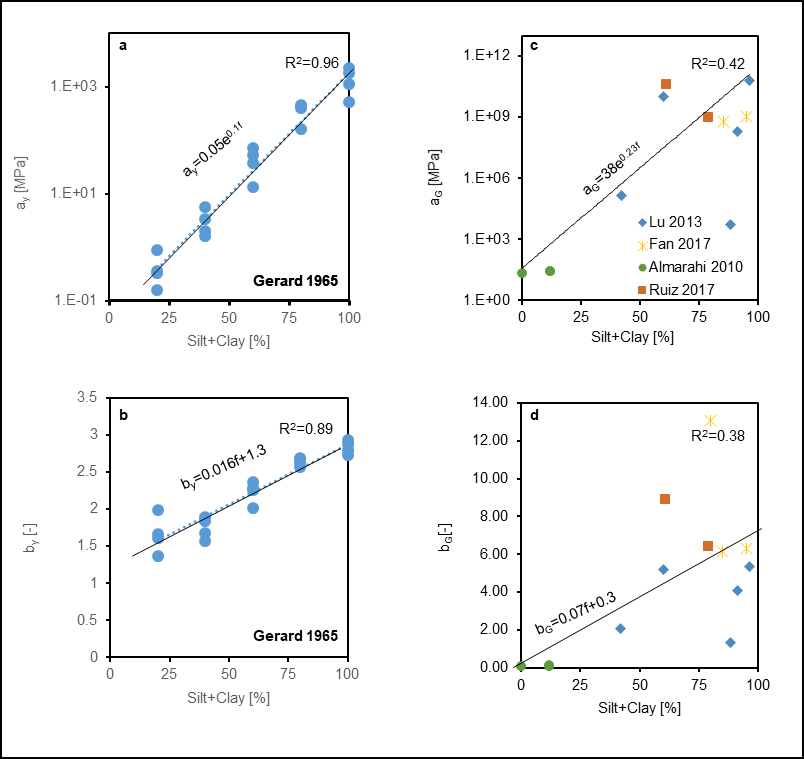


**Supplementary Fig. 1: Soil hydromechanical coefficients as a function of soil fine texture**. Soil shear strength relationships (**a**, **b**) were derived from Gerard *et al.* (1965)^26^. Parameter relationship for shear modulus (**c, d**) to fine texture was derived from data points taken from literature^11, 62-64^.


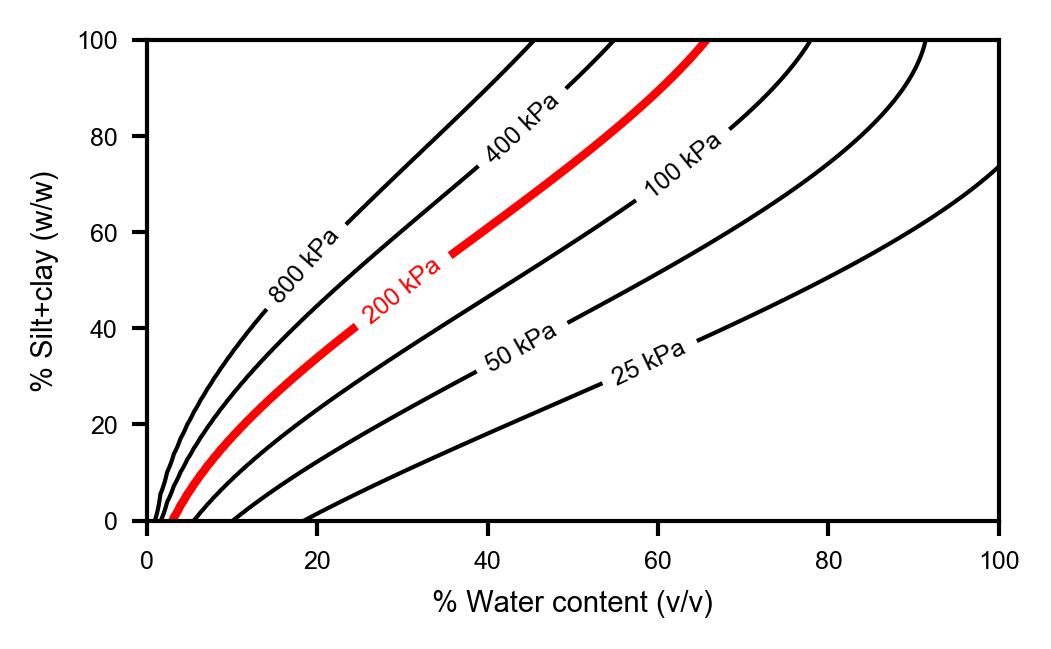


**Supplementary Fig. 2: Profiles of cavity expansion pressures required for given soil texture and soil water contents.** The red curve indicates the earthworm limiting pressures that would hinder bioturbation activity under different texture classes and soil moistures.


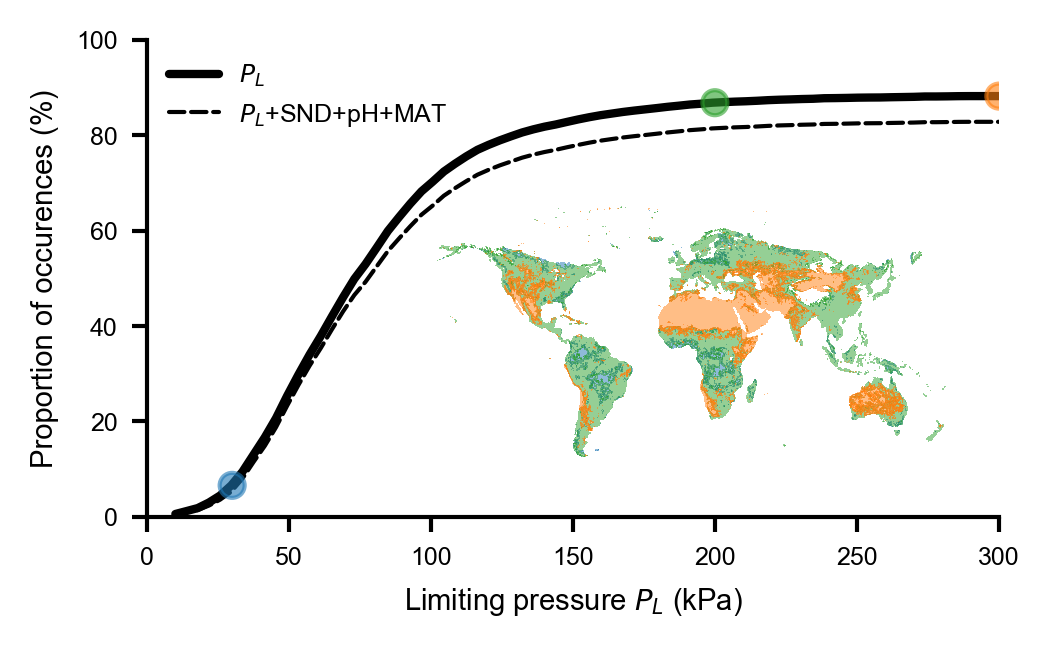


**Supplementary Fig. 3** **Proportion of allotted occurrence as a function of earthworm’s limiting pressure.** 200 kPa was considered the earthworm’s maximum pressure based on experimental measurements^12, 28, 29^. This analysis shows that increasing the pressure threshold to 300 kPa does not increase the proportion of occurrences with the associated areas. Reducing the pressure down to 100 kPa reduces the proportion of occurrence by about 5%. Reductions in pressure below 100 kPa results in a large drop in correspondence with observations.


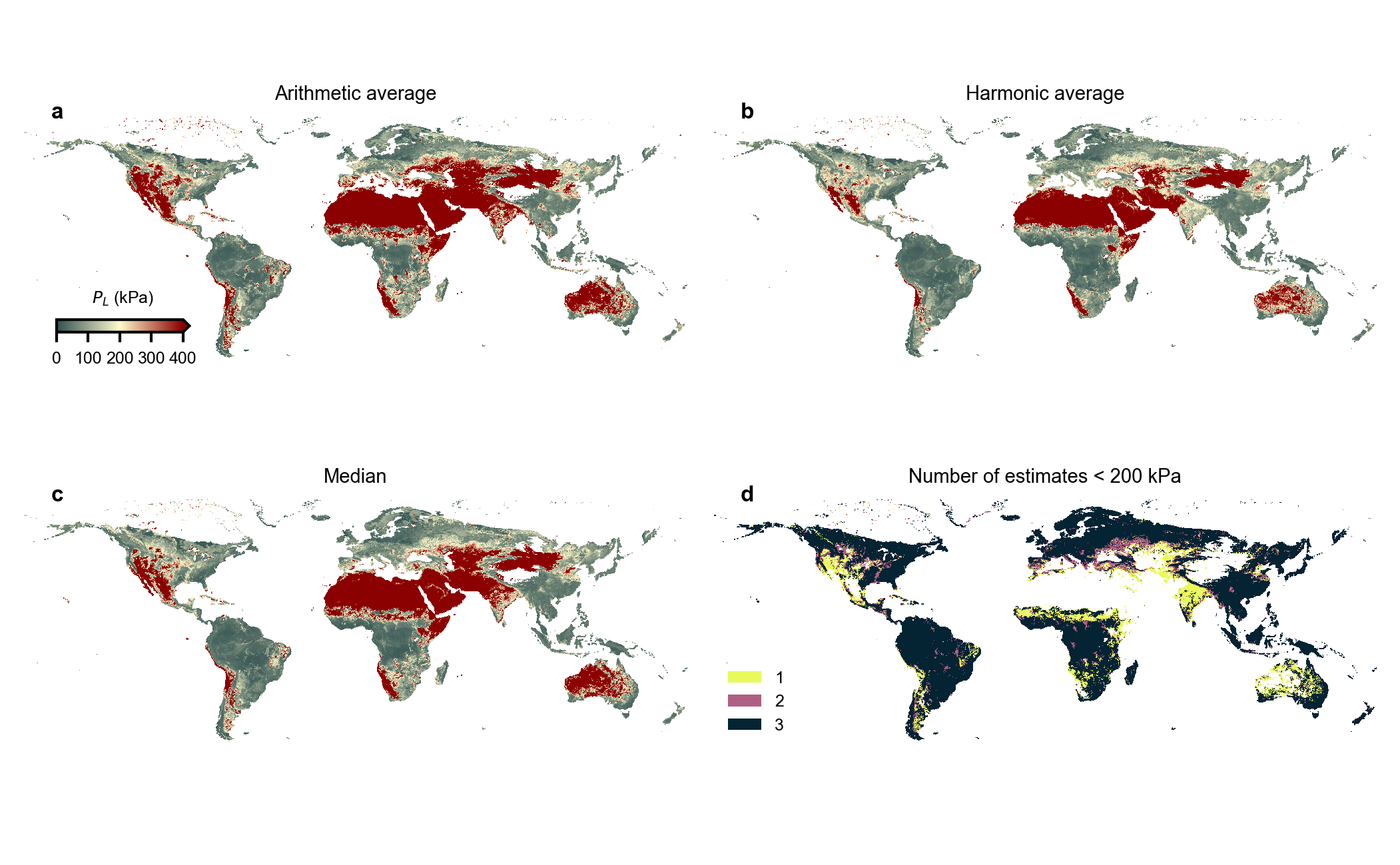


**Supplementary Fig. 4**: **Assessing different averaging techniques.** **a**, Arithmetic mean for limit pressures, **b**, the harmonic mean of limit pressures, and **c**, median values for limit pressures. **d**, provides an estimate for how many times the regions are considered potential earthworm habitats amongst the different techniques (black indicating agreement of all averaging methods considered).


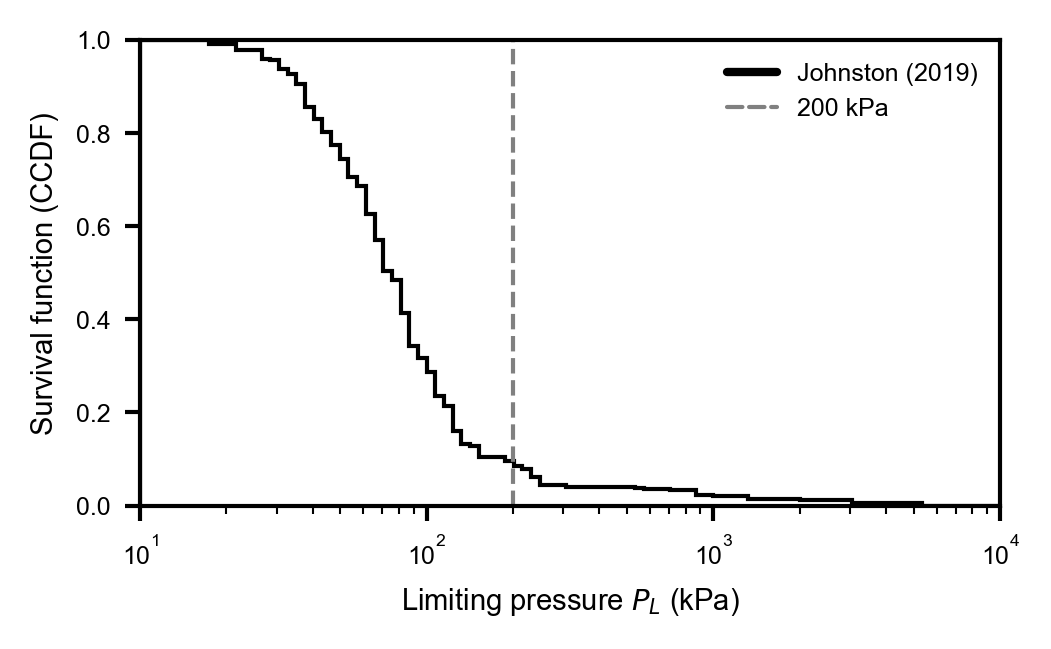


**Supplementary Fig. 5**: **Normalized cumulative distribution of earthworm abundance with average soil limiting pressure magnitudes.** Abundance data was taken from Johnston (2019)^20^ and mapped to limiting pressures using reported geographical coordinates. Over 90% of the earthworms were located in regions with pressures below 200 kPa, which is consistent with the earthworm’s physiological hydroskeletal pressure limit.


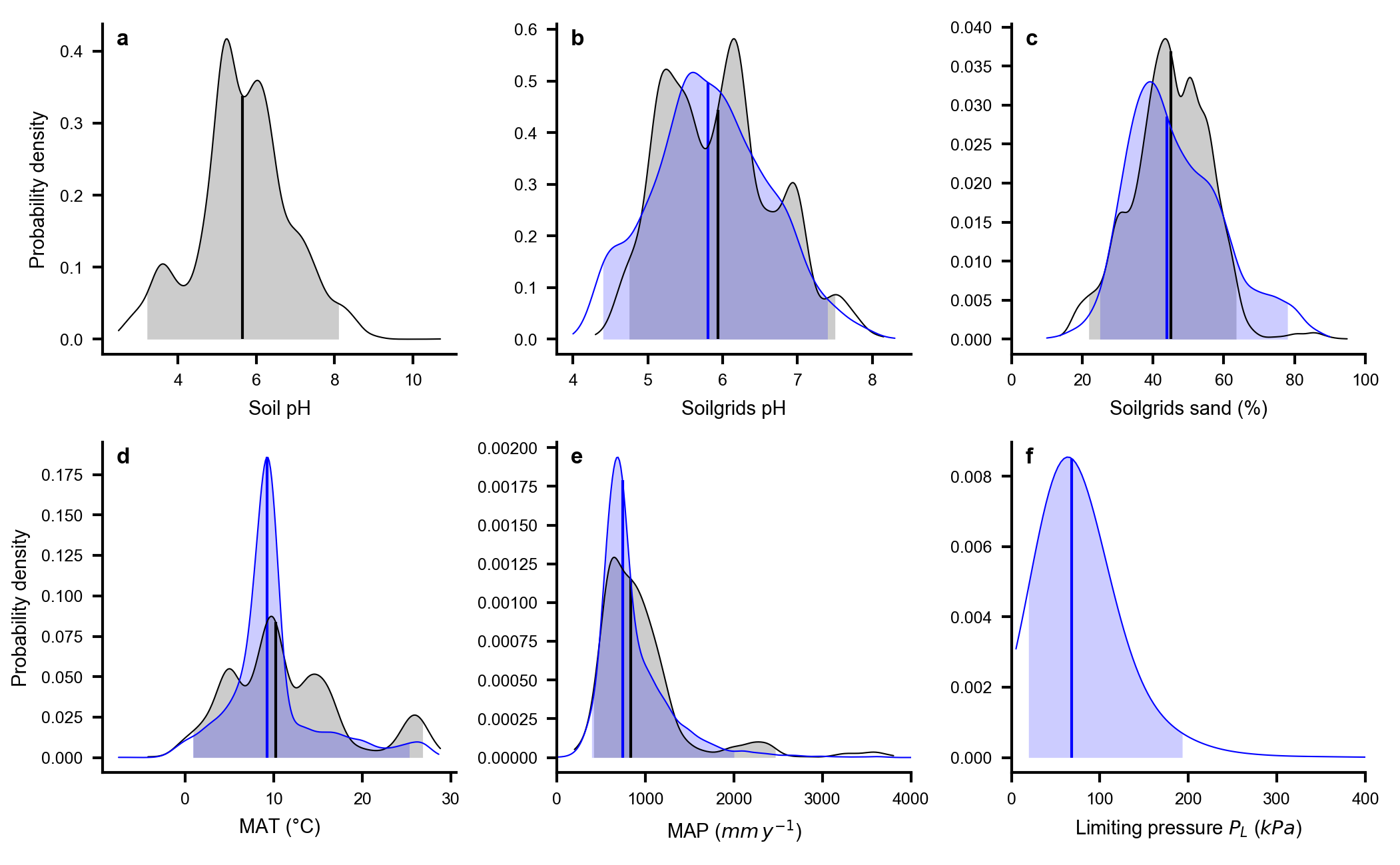


**Supplementary Fig. 6**: **Distribution of additional factors associated with sites of earthworm occurrences.** **a-e**, kernel density estimates for selected variables used to delineate regions of potential earthworm activity. Distributions of occurrences from a recent study^14^ (black) and values at sites used in the current study^15,46^ (blue) are compared. Shaded areas contain 95% of values and the vertical line indicates the median. The recent study^14^ enables comparison of **a**, soil pH measured on-site with **b**, soil pH from SoilGrids as used in our study. The range of SoilGrids^30^ pH values is narrower and most of the occurrences were reported for sites with SoilGrids^30^ pH > 4.5. **c**, Sand content from SoilGrids^30^ at which occurrences were reported. **d**, distribution of mean annual temperature (MAT) and **e**, mean annual precipitation (MAP). **f,** Limiting pressure for earthworm cavity expansion as estimated in this study.


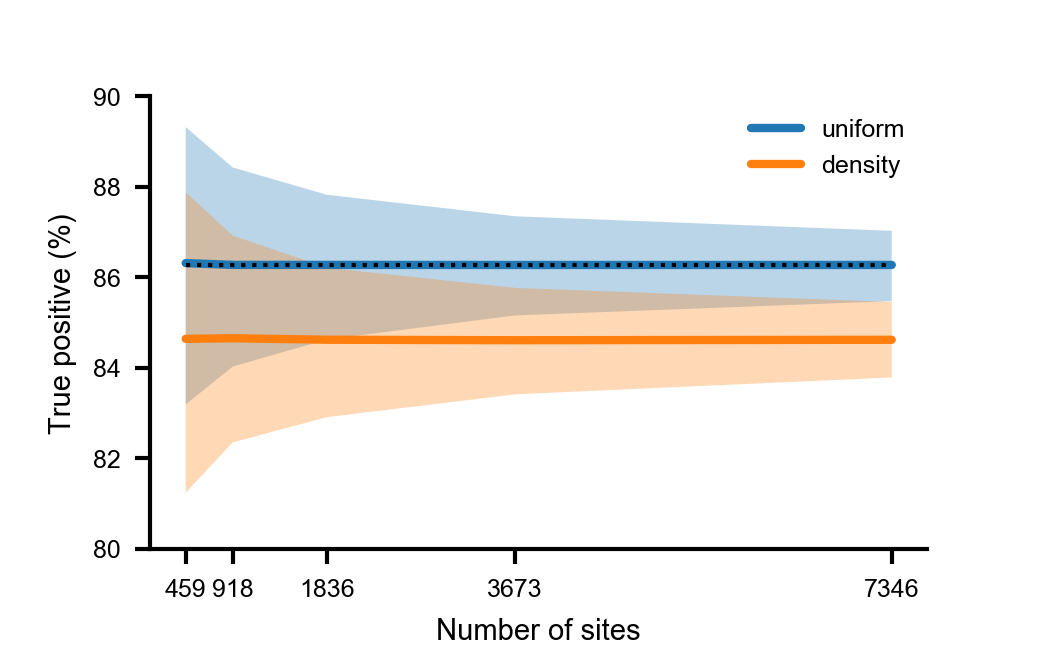


**Supplementary Fig. 7**: **Robustness of true positive rate (hit-rate, sensitivity) under variation of sample size for two sampling schemes.** Random resampling with replacement (n_boot_ = 5000) of sites with earthworm occurrences is shown as solid lines and shading (representing median and central 95%) for two sampling schemes. Sites were selected with uniform probability (blue) or with probabilities inverse to the density of reported occurrences in a five-point neighborhood (orange) thereby penalizing sites with many reported occurrences nearby (attempting to address observational bias). The dashed line represents the hit rate using the full dataset.


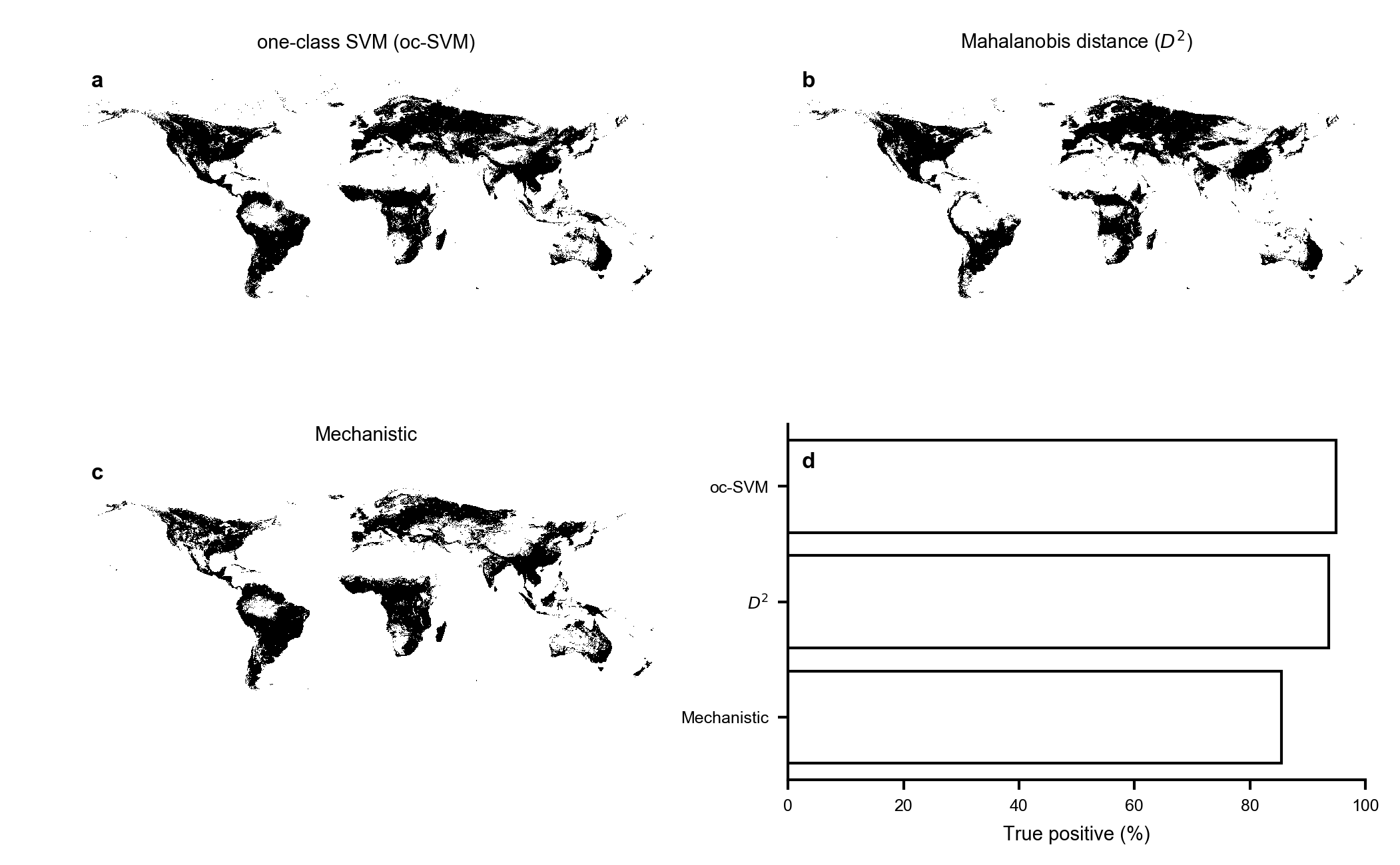


**Supplementary Fig 8**. **Comparing two data-driven approaches to our mechanistic model.** Masks obtained from **a,** one-class support vector machine (oc-SVM)^65^ and **b,** based on the Mahalanobis distance (*D^2^*)^66^ are compared with **c,** our mechanistic model. **d**, The proportion of true positives for each approach.


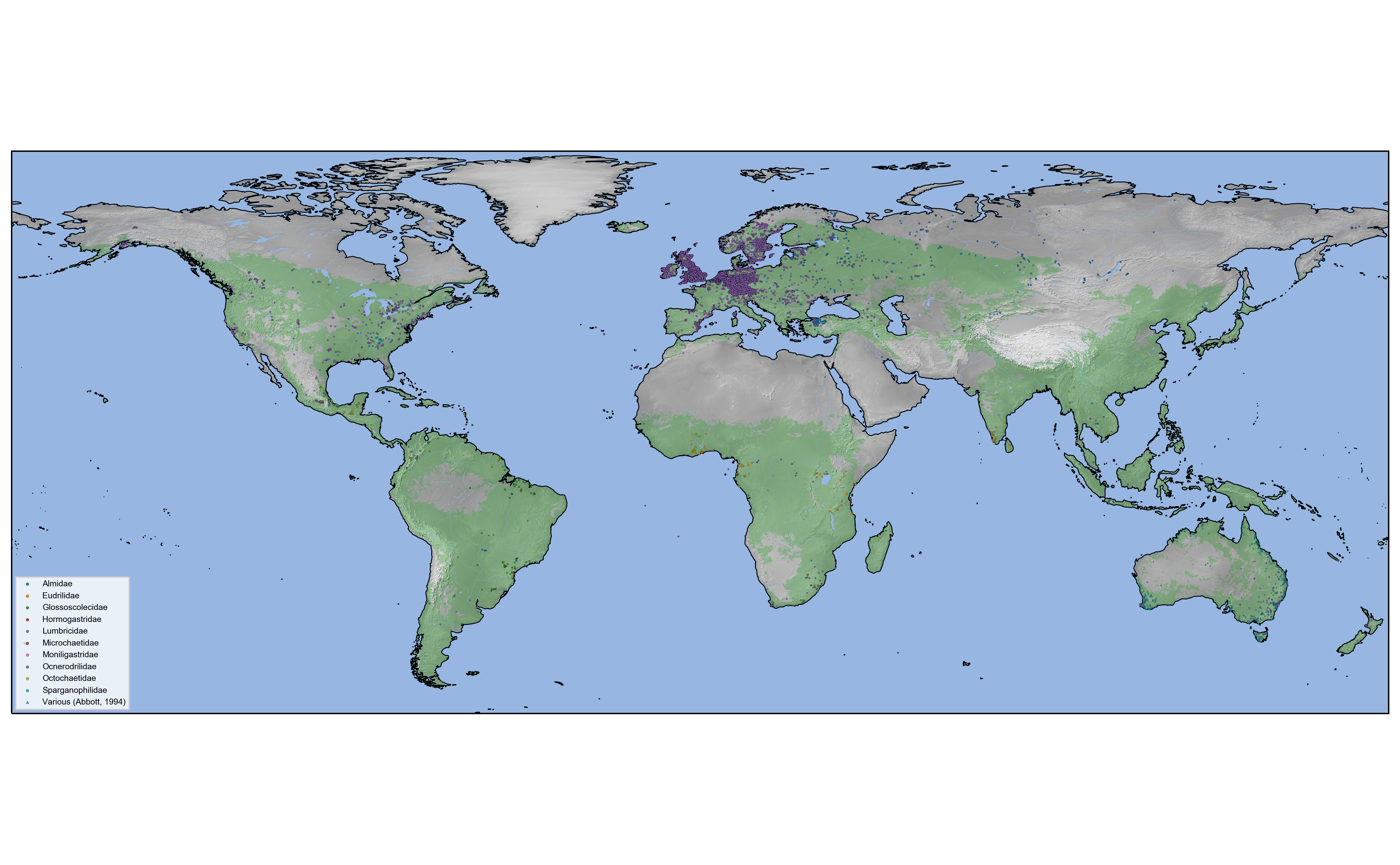


**Supplementary Fig. 9**: **Global distribution of occurrences for ten species obtained from the Global Biodiversity Information Facility and an additional study from Australia^15^.** The green shading indicates the modeled regions that are hospitable to earthworms based on soil mechanics and additional factors. Some occurrence data was monitored on islands smaller than figure resolution. A high-quality version is provided with the supporting data (Supplementary data Fig. S9 - hires.pdf in DOI:10.3929/ehtz-b-000476615^61^ upon publication). Made with Natural Earth. Free vector and raster map data @ naturalearthdata.com.
