# Supplementary material for: Global earthworm distribution and activity windows based on soil hydromechanical constraints
Source: Commun Biol. 2021 May 21;4:612. doi: 10.1038/s42003-021-02139-5 (PMC8140072; doi:10.1038/s42003-021-02139-5)
Supplement: Supplementary file 4 — Reporting Summary [file 42003_2021_2139_MOESM4_ESM.pdf]

nature research

Corresponding author(s): Siul Ruiz

Last updated by author(s): Mar 29, 2020

Reporting Summary

Nature Research wishes to improve the reproducibility of the work that we publish. This form provides structure for consistency and transparency in reporting. For further information on Nature Research policies, see our Editorial Policies and the Editorial Policy Checklist.

Statistics

For all statistical analyses, confirm that the following items are present in the figure legend, table legend, main text, or Methods section.

n/a	Confirmed	
☒	☐	The exact sample size (*n*) for each experimental group/condition, given as a discrete number and unit of measurement
☒	☐	A statement on whether measurements were taken from distinct samples or whether the same sample was measured repeatedly
☒	☐	The statistical test(s) used AND whether they are one- or two-sided<br>*Only common tests should be described solely by name; describe more complex techniques in the Methods section.*
☒	☐	A description of all covariates tested
☐	☒	A description of any assumptions or corrections, such as tests of normality and adjustment for multiple comparisons
☐	☒	A full description of the statistical parameters including central tendency (e.g. means) or other basic estimates (e.g. regression coefficient) AND variation (e.g. standard deviation) or associated estimates of uncertainty (e.g. confidence intervals)
☒	☐	For null hypothesis testing, the test statistic (e.g. *F*, *t*, *r*) with confidence intervals, effect sizes, degrees of freedom and *P* value noted<br>*Give P values as exact values whenever suitable.*
☒	☐	For Bayesian analysis, information on the choice of priors and Markov chain Monte Carlo settings
☒	☐	For hierarchical and complex designs, identification of the appropriate level for tests and full reporting of outcomes
☒	☐	Estimates of effect sizes (e.g. Cohen's *d*, Pearson's *r*), indicating how they were calculated

Our web collection on statistics for biologists contains articles on many of the points above.

Software and code

Policy information about availability of computer code

Data collection	All data used in the study are provided via DOI's from which they were acquired. All bespoke codes will be provided upon request.
Data analysis	Data analysis was conducted in bespoke python scripts. All relevant information is provided in the manuscript text regarding procedure.

For manuscripts utilizing custom algorithms or software that are central to the research but not yet described in published literature, software must be made available to editors and reviewers. We strongly encourage code deposition in a community repository (e.g. GitHub). See the Nature Research guidelines for submitting code & software for further information.

Data

Policy information about availability of data

All manuscripts must include a data availability statement. This statement should provide the following information, where applicable:

- Accession codes, unique identifiers, or web links for publicly available datasets
- A list of figures that have associated raw data
- A description of any restrictions on data availability

All data used in this study is available from public sources ((https://doi.org/10.15468/dl.xstqow, https://doi.org/10.15468/dl.wghggg, https://doi.org/10.15468/dl.3yj8pk, https://doi.org/10.15468/dl.lzuwlg, https://doi.org/10.15468/dl.vwqtsk, https://doi.org/10.15468/dl.brqmht, https://doi.org/10.15468/dl.ghccto, https://doi.org/10.15468/dl.dk97gk, https://doi.org/10.15468/dl.xjw6kc, https://doi.org/10.15468/dl.9a4ojx).). Data underlying maps of potential earthworm habitats will be deposited in a public repository upon publication (meanwhile it is available from the corresponding author upon request).

Field-specific reporting

Please select the one below that is the best fit for your research. If you are not sure, read the appropriate sections before making your selection.

☐ Life sciences ☐ Behavioural & social sciences ☒ Ecological, evolutionary & environmental sciences

For a reference copy of the document with all sections, see nature.com/documents/nr-reporting-summary-flat.pdf

Ecological, evolutionary & environmental sciences study design

All studies must disclose on these points even when the disclosure is negative.

Study description	Earthworms activity modifies soil structure and promotes ecological and hydrological soil functioning. Earthworms use their flexible hydroskeleton to burrow and expand biopores, hence their activity is constrained by soil hydromechanical conditions that permit deformation at earthworm's maximal hydroskeletal pressure (≈ 200 kPa). A novel biophysical model links earthworms' biomechanical limits with bioturbation permitting soil conditions across biomes and climate regions. We inject additional constraints such as freezing temperatures, soil pH, and high sand content that exclude earthworm activity to develop the first predictive global map of earthworm habitats in good agreement with observations. Earthworm activity is strongly constrained by variable seasonal patterns across latitudes. The mechanistic model delineates potential for earthworm migration and regions sensitive to climate and land use changes.
Research sample	No samples were taken for this study.
Sampling strategy	No samples were taken for this study.
Data collection	). For each geographic location we then evaluate the parametrized model using soil textural information from SoilGrids digital soil maps30 and monthly averaged soil moisture estimates from ERA5-land (https://doi.org/10.24381/cds.68d2bb30). All global raster data was harmonized to a common grid of 0.1° resolution (~11 km) using nearest neighbor interpolation of the upper most soil depth layer (0-5cm and 0-7 cm for SoilGrids and ERA-5 land, respectively). The limiting pressure (equation (2)) was calculated for the entire record of the ERA5-land dataset that ranges from 1981 to 2019 at a monthly resolution. We compared our theoretically determined regions with previously published empirical maps that outline earthworm distributions for Australia15 and North America16 and with presence-only data of ten earthworm species (Almidae, Eudrilidae, Glossoscolecidae, Hormogastridae, Lumbricidae, Microchaetidae, Moniligastridae, Ocnerodrilidae, Octochaetidae, Sparganophilidae) as deposited in the Global Biodiversity Information Facility (GBIF) database (https://doi.org/10.15468/dl.xstqow, https://doi.org/10.15468/dl.wghggg, https://doi.org/10.15468/dl.3yj8pk, https://doi.org/10.15468/dl.lzuwlg, https://doi.org/10.15468/dl.vwqtsk, https://doi.org/10.15468/dl.brqmht, https://doi.org/10.15468/dl.ghccto, https://doi.org/10.15468/dl.dk97gk, https://doi.org/10.15468/dl.xjw6kc, https://doi.org/10.15468/dl.9a4ojx).
Timing and spatial scale	All global raster data was harmonized to a common grid of 0.1° resolution (~11 km) using nearest neighbor interpolation of the upper most soil depth layer (0-5cm and 0-7 cm for SoilGrids and ERA-5 land, respectively). The limiting pressure (equation (2)) was calculated for the entire record of the ERA5-land dataset that ranges from 1981 to 2019 at a monthly resolution. Based on the limiting pressure time series, we estimate the number of consecutive months below 200 kPa and the ensemble average pressure for every grid cell. A comparison of averaging methods is reported in the Supplementary Information and we reported harmonic averages throughout the main text. Two specific regions were selected to illustrate temporal activity windows: a grassland located at 9.55oN, 14.65oE and a desert located at -22.95oN, 132.95oE. We aggregated the limiting pressure time series to climatic monthly values and compared with daily climatic precipitation estimates obtained from MSWEP33. Daily precipitation estimates were smoothened using a 30-day rolling average for comparison with monthly pressure values and to delineate time windows of earthworm burrowing activity
Data exclusions	No data was excluded in this study.
Reproducibility	All data used in this study is available from public sources. Data underlying maps of potential earthworm habitats will be deposited in a public repository upon publication (meanwhile it is available from the corresponding author upon request). As such, this will be reproducible.
Randomization	This is not relevant to our study. We base our analysis on a physical model. We are not conducting a comprehensive statistical study.
Blinding	This is not relevant to our study. We base our analysis on a physical model. We are not conducting a comprehensive statistical study.

Did the study involve field work? ☐ Yes ☒ No

Reporting for specific materials, systems and methods

We require information from authors about some types of materials, experimental systems and methods used in many studies. Here, indicate whether each material, system or method listed is relevant to your study. If you are not sure if a list item applies to your research, read the appropriate section before selecting a response.

Materials & experimental systems

n/a	Involved in the study
☒	☐ Antibodies
☒	☐ Eukaryotic cell lines
☒	☐ Palaeontology and archaeology
☒	☐ Animals and other organisms
☒	☐ Human research participants
☒	☐ Clinical data
☒	☐ Dual use research of concern

Methods

n/a	Involved in the study
☒	☐ ChIP-seq
☒	☐ Flow cytometry
☒	☐ MRI-based neuroimaging
